# Severe kodsi grade IV esophageal candidiasis in an apparently immunocompetent patient without oral thrush: A case report

**DOI:** 10.1016/j.idcr.2026.e02789

**Published:** 2026-09-28

**Authors:** Ahmed Muhammad Bashir, Mohamed Abdulkadir Wardhere, Abdisalam Ismail Hassan

**Affiliations:** a Department of Internal Medicine, Somali Sudanese Specialized Hospital, Mogadishu, Somalia; b Research and Education Unit, Mogadishu Virtual Academy, Mogadishu, Somalia; c Department of Gastroenterology and Hepatology, Somali Sudanese Specialized Hospital, Mogadishu, Somalia; d Faculty of Medicine and Health Sciences, Jamhuriya University of Science and Technology, Mogadishu, Somalia

**Keywords:** Candida esophagitis, Esophageal candidiasis, Apparently immunocompetent, Odynophagia, Kodsi classification

## Abstract

**Background:**

Candida esophagitis is the most common fungal infection of the esophagus and typically occurs in patients with impaired immunity or other recognized predisposing conditions. Severe disease in apparently immunocompetent individuals without oral candidiasis is rare and may delay diagnosis.

**Case presentation:**

A 35-year-old previously healthy man presented with a two-month history of progressive odynophagia, cough, and unintentional weight loss. He had no history of diabetes mellitus, malignancy, chronic kidney or liver disease, corticosteroid use, broad-spectrum antibiotic exposure, proton pump inhibitor therapy, smoking, or alcohol consumption. Human immunodeficiency virus serology was nonreactive, fasting blood glucose and glycated hemoglobin were within normal limits, and examination revealed no oral candidiasis. Upper gastrointestinal endoscopy demonstrated extensive confluent whitish plaques with marked mucosal friability and luminal narrowing, consistent with severe Kodsi grade IV esophageal candidiasis on endoscopic appearance. Histopathological confirmation, fungal culture, species identification, and immunological investigations were not performed because of mucosal friability, financial limitations, and restricted diagnostic resources. The patient was treated with intravenous fluconazole and experienced rapid clinical improvement, with sustained symptomatic recovery at two-week follow-up.

**Conclusion:**

This case demonstrates that severe Kodsi grade IV Candida esophagitis may occur in apparently immunocompetent individuals without oral candidiasis or identifiable risk factors. Clinicians should maintain a high index of suspicion for esophageal candidiasis in patients with persistent odynophagia despite the absence of classical predisposing conditions. Prompt endoscopic evaluation and early antifungal therapy are critical for timely diagnosis and prevention of complications, particularly in resource-limited settings where histopathological confirmation may not be feasible.

## Introduction

Candida esophagitis is the most common fungal infection of the esophagus and the leading cause of infectious esophagitis. [1] Although Candida species, particularly Candida albicans, are normal commensals of the gastrointestinal tract, they become pathogenic when host immune defenses or esophageal mucosal integrity are compromised. [2] Within the gastrointestinal tract, the esophagus is the second most commonly affected site of candidal infection after the oropharynx. [3,4] Consequently, CE is most frequently encountered in patients with recognized predisposing conditions, whereas its occurrence in individuals without identifiable risk factors is uncommon and may present a diagnostic challenge.

Numerous factors have been associated with the development of CE, including advanced age, malnutrition, diabetes mellitus, malignancy, chronic debilitating illnesses, and other immunosuppressive conditions, as well as the use of systemic or inhaled corticosteroids, broad-spectrum antibiotics, proton pump inhibitors, immunosuppressive therapies, and certain herbal medications. [[1], [2], [3], [4], [5]] Excessive alcohol consumption has also been implicated as a potential risk factor. [1] Additionally, disorders that impair esophageal clearance or promote luminal stasis, particularly achalasia and other esophageal motility disorders, facilitate Candida overgrowth and increase the risk of esophageal infection. [6]The clinical presentation of CE ranges from nonspecific gastrointestinal symptoms to classic esophageal manifestations, most commonly odynophagia and dysphagia, and may also include epigastric discomfort, weight loss, malnutrition, or features related to underlying predisposing conditions. The diagnosis is mainly based on characteristic endoscopic findings and is confirmed by histopathological evidence of yeast and hyphal invasion of the superficial esophageal epithelium. [1] Endoscopic severity is commonly graded using the Kodsi classification, where grade I has a few raised white plaques up to 2 mm, grade II has multiple raised white plaques larger than 2 mm without ulceration, grade III has confluent linear and nodular elevated plaques, and grade IV has extensive grade III lesions with marked mucosal friability and occasional narrowing of the esophageal lumen. [7]

Although CE is well recognized in patients with established predisposing conditions, severe Kodsi grade IV disease occurring in individuals without identifiable risk factors is rarely reported. Furthermore, the absence of concomitant oral candidiasis may delay clinical recognition, emphasizing the necessity of considering CE in the differential diagnosis of persistent odynophagia even in apparently immunocompetent patients. We report a case of severe Kodsi grade IV Candida esophagitis in a 35-year-old man without identifiable predisposing conditions or oral candidiasis, highlighting the diagnostic challenges encountered in a resource-limited setting.

## Case presentation

A 35-year-old male farmer presented with a two-month history of dysphagia, progressive odynophagia, cough, and unintentional weight loss. He had no known history of diabetes mellitus, malignancy, chronic kidney disease, chronic liver disease, or any other chronic medical condition. There was no recent exposure to systemic or inhaled corticosteroids, broad-spectrum antibiotics, or proton pump inhibitors. He was a nonsmoker and denied alcohol consumption.

On physical examination, the patient appeared ill. Examination of the oral cavity revealed no oral thrush or other visible candidal lesions. The remainder of the physical examination was unremarkable.

Serological testing for human immunodeficiency virus (HIV) was nonreactive. Screening tests for hepatitis B virus and hepatitis C virus were also negative. Fasting blood glucose and glycated hemoglobin (HbA1c) were within their respective reference ranges, making previously undiagnosed diabetes mellitus unlikely. The erythrocyte sedimentation rate was normal. Chest radiography demonstrated no pulmonary infiltrates, cavitation, masses, or other abnormalities.

Because of persistent dysphagia, odynophagia, and weight loss, upper gastrointestinal endoscopy was performed. The esophageal mucosa was extensively covered by confluent, elevated whitish plaques associated with marked mucosal erythema, friability, focal contact bleeding, and narrowing of the esophageal lumen. These findings were consistent with severe Kodsi grade IV esophageal candidiasis (Fig. 1 A and B).

**Fig. 1:**
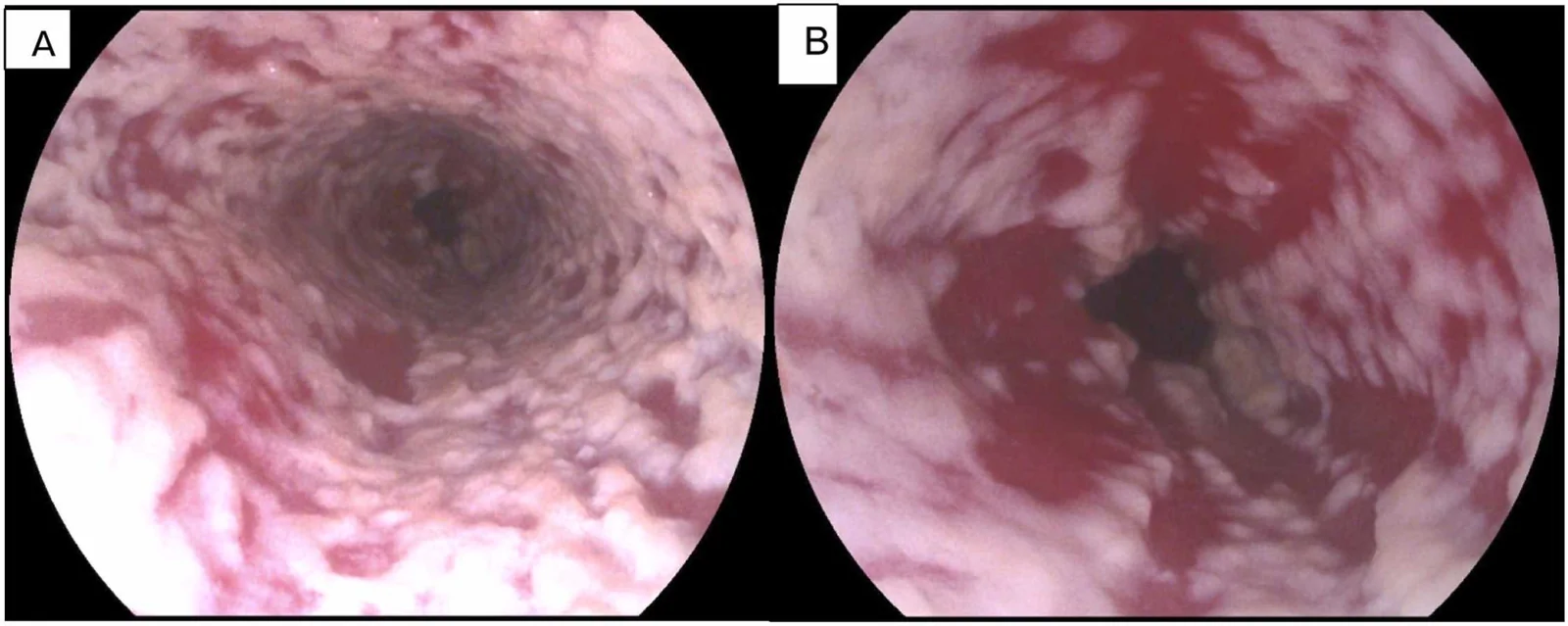
Endoscopic appearance of Kodsi grade IV esophageal candidiasis. (A) Extensive circumferential and confluent whitish plaques coating the esophageal mucosa, with marked luminal narrowing. (B) Severe mucosal erythema and friability with adherent plaques, focal bleeding, and luminal compromise, consistent with advanced esophageal candidiasis.

The stomach demonstrated diffuse gastritis. Gastric mucosal biopsy showed no significant histopathological abnormality and was negative for Helicobacter pylori. No esophageal biopsy or brush cytology was obtained because of the marked mucosal friability and concern for procedure-related complications. In addition, fungal microscopy, fungal culture, species identification, and antifungal susceptibility testing were unavailable because of limited diagnostic resources. CD4 lymphocyte count, assessment for primary or secondary immunodeficiency, and comprehensive nutritional assessment were not performed. Therefore, although no recognized immunosuppressive condition was identified clinically, underlying immune dysfunction or malnutrition could not be completely excluded.

Based on the characteristic endoscopic findings and clinical presentation, a presumptive diagnosis of severe Kodsi grade IV esophageal candidiasis was made. The differential diagnosis included viral esophagitis, reflux esophagitis, medication-induced injury, eosinophilic esophagitis, and esophageal malignancy. These were considered less likely because the extensive adherent confluent whitish plaques, marked friability, and contact bleeding were more characteristic of Candida infection, while the patient lacked relevant immunosuppression, medication exposure, or a discrete esophageal mass. The rapid clinical response to fluconazole further supported the diagnosis. Definitive exclusion of these alternatives was limited by the absence of esophageal biopsy and microbiological testing.

The patient was initially treated with intravenous fluconazole 400 mg once daily for seven days because of severe odynophagia and dysphagia, together with intravenous esomeprazole 40 mg once daily for the associated gastritis and possible reflux-related mucosal irritation. He was subsequently transitioned to oral fluconazole to complete a total treatment duration of 21 days. He demonstrated substantial improvement in dysphagia, odynophagia, and overall clinical condition within one week of therapy. At the two-week follow-up visit, the clinical improvement was sustained. Follow-up endoscopy was performed at one month and demonstrated complete resolution of the previously described esophageal lesions. Clinical follow-up at six months revealed no recurrence of symptoms, and he remained clinically well.

## Discussion

Candida esophagitis (CE) is an opportunistic fungal infection that predominantly affects individuals with impaired immunity or disruption of normal esophageal defense mechanisms. The majority of cases occur in the presence of recognized predisposing factors, including diabetes mellitus, malignancy, corticosteroid exposure, broad-spectrum antibiotic use, proton pump inhibitor therapy, malnutrition, and esophageal motility disorders. [[1], [2], [3], [4], [5], [6]] In contrast, severe CE in patients without identifiable risk factors is uncommon and presents a significant diagnostic challenge. Our patient had no history of diabetes mellitus, chronic kidney or liver disease, malignancy, corticosteroid or antibiotic exposure, proton pump inhibitor use, smoking, or alcohol consumption. Serological testing for human immunodeficiency virus was nonreactive, and both fasting blood glucose and glycated hemoglobin were within normal limits, supporting the absence of common predisposing conditions.

Although Candida albicans commonly colonizes the upper gastrointestinal tract, progression from colonization to invasive esophageal infection generally requires impairment of host immune defenses or disruption of esophageal mucosal integrity. [2] The development of severe disease in a patient without identified immunosuppressive conditions suggests that subtle or unrecognized abnormalities in immune function or esophageal barrier defenses may have contributed. However, the patient should be regarded as apparently immunocompetent rather than definitively immunocompetent, as CD4 lymphocyte count, evaluation for primary or secondary immune defects, and comprehensive nutritional assessment were not performed. Therefore, occult immune dysfunction or malnutrition cannot be completely excluded.

Clinically, the patient presented with progressive odynophagia, cough, and weight loss, findings consistent with the typical manifestations of CE. [3]A notable feature was the absence of oral candidiasis. Although oral thrush commonly accompanies esophageal candidiasis, its absence does not exclude esophageal involvement. Oral thrush may be a useful clinical marker of esophageal candidiasis when present, while esophageal candidiasis can also occur without concomitant oral thrush. [[8], [9], [10], [11]] Therefore, in our patient, the absence of oral lesions did not exclude Candida esophagitis, particularly given the characteristic endoscopic findings and compatible symptoms.

Upper gastrointestinal endoscopy remains the cornerstone of diagnosis, typically demonstrating adherent white plaques or pseudomembranous lesions overlying erythematous and friable esophageal mucosa. [1,4] Although these characteristic endoscopic findings are highly suggestive of esophageal candidiasis, they are not entirely specific and may overlap with other causes of esophageal mucosal injury; therefore, histopathological or cytological confirmation is recommended when feasible. [1,3] The reported prevalence of endoscopically diagnosed esophageal candidiasis ranges from 1% to 8% and has declined since the introduction of newer antifungal agents in the 1990s. [12] In our patient, endoscopy demonstrated extensive confluent white plaques with marked mucosal friability and luminal narrowing, consistent with Kodsi grade IV disease. According to Kodsi's classification, grade IV represents the most advanced stage and reflects extensive mucosal involvement. [7] Most published cases involving apparently immunocompetent individuals describe mild to moderate disease, whereas severe grade IV esophagitis is rarely reported. The presence of luminal narrowing in our patient further suggests advanced inflammatory involvement and underscores the potential for delayed diagnosis when classical risk factors are absent.

The diagnosis was considered presumptive, based on the characteristic endoscopic findings, compatible clinical presentation, and rapid clinical response to fluconazole. Histopathological confirmation was not obtained because esophageal biopsy and brush cytology were deferred owing to marked mucosal friability, the potential risk of perforation, and the patient's financial constraints. Likewise, fungal microscopy, culture, species identification, and antifungal susceptibility testing were unavailable because of limited diagnostic resources. Although histopathological demonstration of fungal invasion remains the diagnostic gold standard, the combination of characteristic endoscopic findings and compatible clinical features provided strong supportive evidence when tissue confirmation was not feasible. The favorable clinical response following combined antifungal and acid-suppressive therapy supported the clinical diagnosis, although the contribution of acid suppression to symptomatic improvement cannot be completely excluded. [13,14]

Several reports have described esophageal candidiasis in immunocompetent or apparently immunocompetent individuals, although the clinical presentation and potential contributing factors vary. Liang et al. reported fungal esophagitis associated with a giant gastric ulcer in an immunocompetent patient, while Narang et al. described recurrent Candida-associated esophageal strictures in an initially immunocompetent patient. [2,5] Abdelfattah and Mahgoub similarly reported severe Candida-associated esophageal stricture in a non-HIV patient without conventional immunosuppression. [4] These reports demonstrate that Candida esophagitis can occasionally cause severe or structural disease outside the classical immunocompromised population.

Pediatric reports further highlight the range of potential contributing factors. Balamtekin et al. described a 13-year-old adolescent with esophageal candidiasis and chronic gastroesophageal reflux, with normal immune evaluation and no significant medication exposure apart from antacids. Endoscopy showed esophageal exudate, erythema, and early ulceration, with histopathological confirmation of Candida. [15]Similarly, Hassan and Mahony reported a 9-year-old girl with esophageal candidiasis, no oral thrush, and normal immunological and HIV evaluations. However, she had received inhaled beclometasone for four years, which was considered a potential predisposing factor; endoscopy showed Candida plaques and mucosal erythema, with biopsy confirming Candida hyphae. [16]Esophageal candidiasis has also been reported in other apparently immunocompetent settings. Greenspoon and Kivnick described invasive *Candida albicans* esophagitis in a pregnant woman without oral thrush, with negative HIV testing and normal T-cell profiles. Candida was confirmed by culture and histopathology, although pregnancy, gestational diabetes, and cimetidine exposure were considered potential contributing factors. [17] Another report described concomitant Candida and herpes simplex esophagitis in a previously healthy adult without conventional immunosuppressive conditions. [18]

Collectively, these reports indicate that esophageal candidiasis may occur in patients without recognized immunosuppressive conditions, potentially in association with impaired mucosal integrity, gastroesophageal reflux, corticosteroid exposure, acid-suppressive therapy, pregnancy-related physiological changes, or other incompletely recognized factors. In contrast to many previously reported cases, our patient had severe Kodsi grade IV disease, characterized by extensive confluent plaques, marked mucosal friability, contact bleeding, and luminal narrowing, without oral thrush or an identified conventional predisposing factor. Although histopathological and microbiological confirmation was not feasible, the characteristic endoscopic findings, compatible clinical presentation, favorable response to treatment, and complete endoscopic resolution at one-month follow-up provided strong supportive evidence for the presumptive diagnosis. Thus, this case adds to the limited literature by illustrating that severe endoscopically classified Candida esophagitis can occur in an apparently immunocompetent adult without oral candidiasis or an identifiable conventional risk factor.

This case also highlights the diagnostic challenges encountered in resource-limited settings. The lack of histopathology, fungal culture, species identification, antifungal susceptibility testing, and comprehensive immunological investigations limited definitive microbiological confirmation and detailed assessment of the patient's immune status. Nevertheless, the characteristic endoscopic findings, exclusion of common predisposing conditions, compatible clinical presentation, and favorable therapeutic response provided compelling evidence supporting the diagnosis.

In conclusion, this case emphasizes several important clinical lessons. Severe Candida esophagitis should remain in the differential diagnosis of persistent odynophagia, even in patients without recognized risk factors or oral candidiasis. The absence of oral thrush does not exclude significant esophageal involvement, and early endoscopic evaluation is essential for timely diagnosis and initiation of antifungal therapy, potentially preventing complications such as esophageal stricture and persistent luminal narrowing.

## Conclusion

Severe Candida esophagitis can occur in apparently immunocompetent individuals without identifiable predisposing factors or concomitant oral candidiasis. This case highlights that the absence of classical risk factors should not preclude consideration of esophageal candidiasis in patients presenting with persistent odynophagia or dysphagia. Characteristic endoscopic findings, together with exclusion of common underlying conditions and a favorable response to antifungal therapy, may support a presumptive diagnosis when histopathological confirmation is not feasible, particularly in resource-limited settings. Early recognition and prompt initiation of antifungal treatment are essential to prevent disease progression and complications such as esophageal stricture.

## CRediT authorship contribution statement

**Ahmed Muhammad Bashir:** Conceptualization, Writing – original draft, Writing – review & editing. **Mohamed Abdulkadir Wardhere:** Investigation, Writing – review & editing. **Abdisalam Ismail Hassan:** Writing – review & editing, Supervision.

## Ethical approval and consent to participate

Ethical approval was not required for this case report according to the institutional policies of our hospital. Written informed consent was obtained from the patient to collect and use the clinical information for publication. All identifying information has been anonymized to protect the patient's privacy.

## Ethics declaration

Written informed consent to take part in the study and to publish the article has been obtained from all participants or their legal representatives. The privacy rights of participants have been observed.

This study included organ or tissue donors. This study includes human biological material and consent was obtained by donors, or their next of kin or legal representatives, for use in this study and for publication of the article. The samples used in this research were not sourced from executed prisoners or prisoners of conscience.

This study was performed in compliance with relevant laws, regulatory frameworks and guidelines where the research took place. The Somali Sudanese Specialized Hospital granted an exemption. The authors provided the following explanation: Ethical approval was not required for this case report according to the institutional policies of our hospital. Written informed consent was obtained from the patient to collect and use the clinical information for publication. All identifying information has been anonymized to protect the patient's privacy

This research follows the CARE guidelines and the CARE checklist.

## Consent for publication

Written informed consent was obtained from the patient for publication of this case report and any accompanying images. A copy of the written consent is available for review by the Editor-in-Chief of this journal upon reasonable request.

## Funding

The authors received no financial support for the research, authorship, or publication of this article.

## Declaration of Competing Interest

The authors declare that they have no known competing financial interests or personal relationships that could have appeared to influence the work reported in this paper

## Data Availability

All data generated or analyzed during this study are included in this published article. Additional information is available from the corresponding author upon reasonable request.
